# Structures of ACE2–SIT1 recognized by Omicron variants of SARS-CoV-2

**DOI:** 10.1038/s41421-022-00488-x

**Published:** 2022-11-16

**Authors:** Yaping Shen, Jianhui Wang, Yaning Li, Yuanyuan Zhang, Ruilin Tian, Renhong Yan

**Affiliations:** 1 Center for Infectious Disease Research, Westlake Laboratory of Life Sciences and Biomedicine, Key Laboratory of Structural Biology of Zhejiang Province, School of Life Sciences, Westlake University, Hangzhou, Zhejiang Province China; 2 School of Medicine, Southern University of Science and Technology, Shenzhen, Guangdong Province China; 3 Key University Laboratory of Metabolism and Health of Guangdong, Southern University of Science and Technology, Shenzhen, Guangdong Province China; 4 Beijing Advanced Innovation Center for Structural Biology, Tsinghua-Peking Joint Center for Life Sciences, School of Life Sciences, Tsinghua University, Beijing, China

**Keywords:** Cryoelectron microscopy, Mechanisms of disease

Dear Editor,

The continued spread of severe acute respiratory syndrome coronavirus 2 (SARS-CoV-2) Omicron variants has caught another wave of COVID-19 pandemic and raised great public health and economic concerns^1,2^. The Omicron BA.2 subvariant has quickly outcompeted the BA.1 subvariant since Feb of 2022^3^. And now, the BA.4/BA.5 subvariants, which share the same S protein mutations, displayed a higher transmission advantage than BA.2 and exhibit more powerful immune evasion^4^. Angiotensin-converting enzyme 2 (ACE2) is the critical cellular receptor for SARS-CoV-2, directly binding with the receptor binding domain (RBD) of spike glycoprotein (S protein)^5–8^. The full-length ACE2 comprises an N-terminal peptidase domain (PD) and a C-terminal collectrin-like domain (CLD) that contains a single transmembrane helix (TM) and a short intracellular segment^9,10^. The PD of ACE2 is the target of SARS-CoV-2 S protein and also mediates the maturation of angiotensin (Ang) which controls vasoconstriction and blood pressure^11^. The CLD of ACE2 is reported as the chaperone for membrane trafficking of amino acid transporter B^0^AT1 (*SLC6A19*) and SIT1 (*SLC6A20*)^12^. Recently, a genome-wide association study (GWAS) revealed that variants at the 3p21.31 locus containing regulatory region of *SLC6A20* are closely associated with the risk and severity of COVID-19 infection^13^. Deletion of this locus reduces the expression level of *SLC6A20*, suggesting that *SLC6A20* might be one of the potential causal genes responsible for COVID-19 risk^14^. However, whether and how SIT1 affects the recognition of ACE2 by SARS-CoV-2 remain unclear. Here, we report the cryo-EM structures of the full-length human ACE2 bound to the RBD of the SARS-CoV-2 Omicron subvariants BA.2 and BA.4/BA.5 (hereafter referred to as BA.5) at an overall resolution of 3.1 Å and 3.2 Å in the presence of SIT1, respectively. Pairwise comparison reveals a number of variations that may determine the different affinities between ACE2 and the RBDs from different SARS-CoV-2 variants.

To investigate the role of SIT1 involved in COVID-19, we first isolated the ACE2–SIT1 complex. Full-length human His-tagged ACE2 and Flag-tagged SIT1 were co-expressed in human embryonic kidney (HEK) 293 F cells. After tandem affinity purification and size exclusion chromatography (SEC), the complex exhibits a single monodisperse peak, suggesting high homogeneity (Supplementary Fig. S1a).

To reveal the interaction details between ACE2–SIT1 and the SARS-CoV-2 Omicron variants, the RBD from SARS-CoV-2 BA.2 and BA.5 subvariants, respectively, was mixed with ACE2–SIT1 complex at a stoichiometric ratio of ~2.4:1 for 30 min and applied to SEC to remove excess RBD (Fig. 1a; Supplementary Fig. S2a). The ternary complex exhibits high homogeneity and the peak fractions containing the complex were concentrated for further cryo-EM sample preparation and structure determination.

**Fig. 1:**
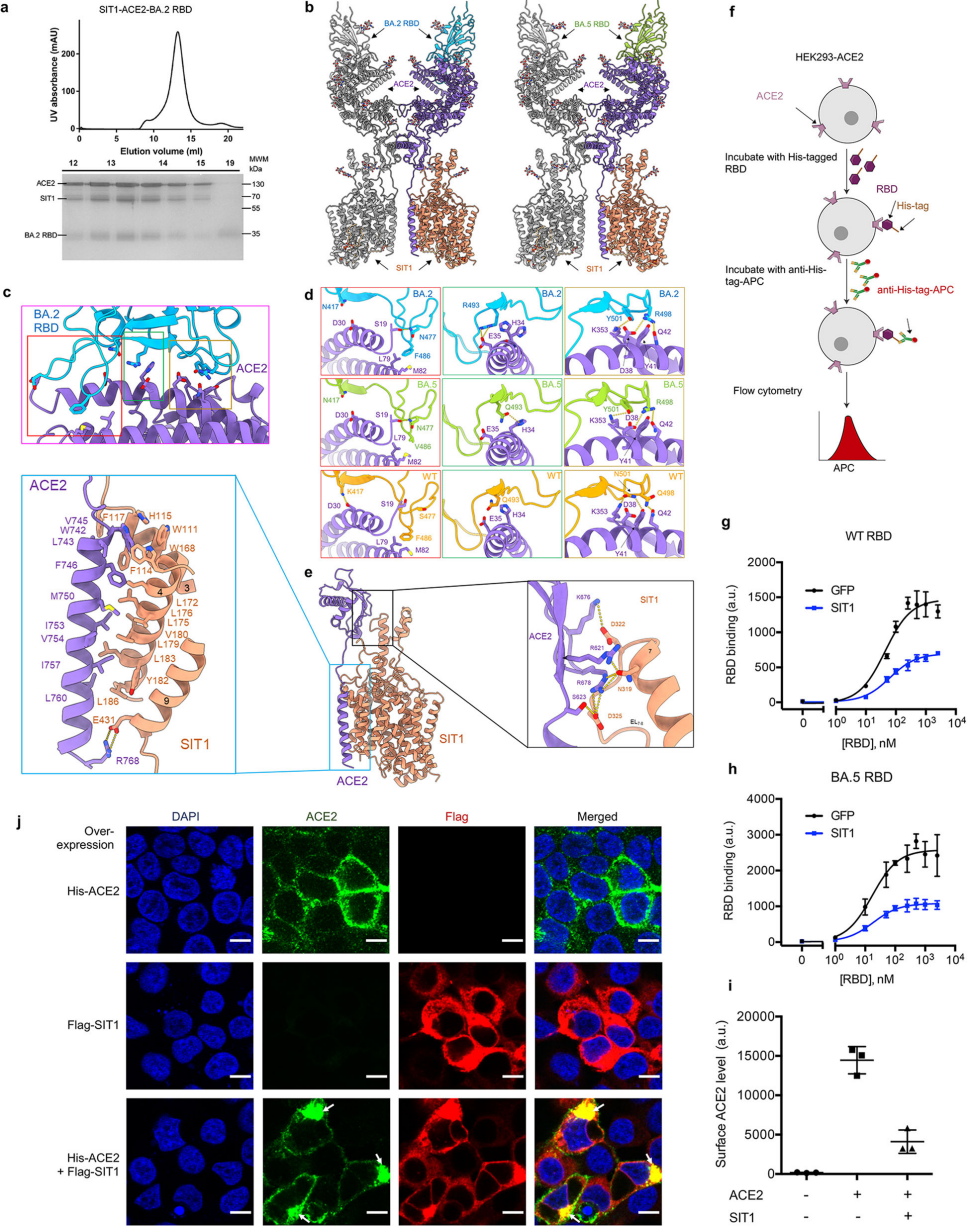
Characterization of the RBD–ACE2–SIT1 complex. **a** Representative SEC purification of the SIT1–ACE2–BA.2 RBD. SDS-PAGE was visualized by Coomassie blue staining. **b** Overall structure of the SIT1–ACE2–BA.2 RBD (left) complex and SIT1–ACE2–BA.5 RBD complex (right). The complexes are colored by subunits. Promoters are shown in different colors. **c** Interface of ACE2 and BA.2 RBD. **d** The detailed analysis of the interface between Omicron variants and ACE2. RBD of BA.2, BA.5, and WT are colored blue, green, and orange, respectively. The PDB ID for the WT structure is 6M17. **e** Structure of the transmembrane domain of ACE2 and SIT1 is shown in the middle. ACE2 and SIT1 are colored purple and light salmon, respectively. Insets: enlarged views of the interface between the transmembrane helix of ACE2 and transmembrane helix 3, 4, 9 of SIT1 (left) and the interface between the extracellular loop of ACE2 and TM7 of SIT1 (right). **f** Diagram of the flow cytometry-based RBD binding assay. See Methods for detail. **g**, **h** RBD binding curves of HEK293-ACE2 cells overexpressing GFP (black) or SIT1(blue) for WT (**g**) and BA.5 RBD (**h**). For WT RBD, EC_50_ values for cells overexpressing GFP and SIT1 are 43.64 nM and 63.23 nM, respectively. For BA.5 RBD, EC_50_ values for cells overexpressing GFP and SIT1 are 16.99 nM and 18.41 nM, respectively. Data are shown as means ± SD (*n* = 3). **i** Cell-surface ACE2 levels of cells overexpressing ACE2 and/or SIT1 measured by flow cytometry. Data are shown as means ± SD (*n* = 3). **j** Representative confocal images for ACE2 and SIT1 localization in cells overexpressing ACE2 and/or SIT1. White arrows indicate the colocalization of ACE2 and SIT1 in the cytosol. Scale bar, 10 µm.

The cryo-EM structures of Omicron BA.2/5 bound with ACE2–SIT1 complex were determined at an overall resolution of 3.1 Å and 3.2 Å, respectively (Fig. 1b; Supplementary Figs. S1–S5 and Table S1). The interfaces between RBD and ACE2 were further improved to 3.0 Å and 3.1 Å, respectively, with the focused refinement, supporting reliable modelling and interface analysis.

From an overall view, the RBD binds to the ACE2–SIT1 complex with a ratio of 2:2:2. Each PD binds to one RBD. The interface between RBD and ACE2-PD for BA.2 and BA.5 is almost consistent except that Arg493 of BA.2 forms a new salt bridge with Glu35 of ACE2, whereas Wildtype (WT) and BA.5 retain Gln493 (Fig. 1c, d; Supplementary Fig. S6). When compared with WT, the markable difference is that the Asn477 and Arg498 of BA.5 form new polar interactions with Ser19 and Asp38 of ACE2, respectively (Fig. 1d). The loss of hydrogen-bond (H-bond) was found between Tyr501 of BA.5 RBD and Tyr41 of ACE2 when compared with WT RBD, and K417N mutation disrupts the original interaction with Asp30 of ACE2 (Fig. 1d). Additionally, Val486 of BA.5 retains hydrophobic interactions with the Leu79 and Met82 of ACE2. Taken together, these mutations of Omicron subvariants remodeled the interaction net between RBD and ACE2.

To investigate the binding characteristics of Omicron subvariants, we measured the binding affinities between RBD of WT, BA.1, BA.2, BA.5 and the PD of ACE2 using Bio-Layer Interferometry (BLI). The BA.2 RBD and BA.5 RBD bind to secreted form of PD with *K*_D_ of 4.15 ± 0.01 nM and 4.55 ± 0.03 nM, respectively; the interactions are about 4 folds stronger than that between WT RBD and PD (*K*_D_ = 18.4 ± 0.03 nM) (Supplementary Fig. S7). These results are consistent with a previous report^15^. This indicates that the newly formed polar interactions and salt bridges in Omicron variants may not only neutralize the lost interactions but also improved the binding affinity.

Understanding the interaction between ACE2 and SIT1 might help to uncover the role of SIT1 in COVID-19. We mainly focused on the detailed interface analysis in BA.2 RBD–ACE2–SIT1 structure since the interfacial resolution of ACE2 and SIT1 reached 3.4 Å. ACE2 interacts with SIT1 extensively via the extracellular region and the membrane region (Fig. 1e). On the extracellular side, the remarkably extended TM7 and nearby segments of SIT1 are connected to the neck domain of ACE2. The CLD of ACE2 interacts with SIT1 at the C-terminal end of TM7 and the following Extracellular Loop 7–8 (EL_7–8_) mainly through hydrophilic interactions. The Asn319 of SIT1 is H-bonded with Arg621 and Arg678 of ACE2. The Asp322 and Asp325 of SIT1 form salt bridges with Lys676 and Arg678, respectively (Fig. 1e, right panel). In the membrane region, the TM of ACE2 interacts with TM3 and TM4 of SIT1 through a patch of hydrophobic residues (Fig. 1e, left panel). Notably, the Trp742 of ACE2 and Trp111, His115, and Trp168 of SIT1 are involved in a π–π interaction net, which might further help to maintain proper conformation of ACE2 on the membrane. Besides, the Arg768 at the C-terminal end of TM of ACE2 forms salt bridge with Glu431 on TM9 of SIT1.

We also compared the ACE2–SIT1 complex with the previously reported ACE2–B^0^AT1 complex and found that they exhibit quite a similar binding pattern (Supplementary Fig. S8). When compared to dDAT and other LeuT-fold transporters, TM7 of SIT1 or B^0^AT1 is particularly long with its C-terminus extruding out of the membrane and connected with ACE2 (Supplementary Fig. S8). These structure-based analyses indicate that ACE2–SIT1 complex are tightly coupled through the connection between TM7 of SIT1 and the neck domain of ACE2, and the π–π interaction net on the membrane region.

We next determined if the interaction between ACE2 and SIT1 can modulate SARS-CoV-2 RBD binding to human cells. Using HEK293 cell line overexpressing hACE2 (HEK293-ACE2), we measured the binding of WT and BA.5 RBDs to cells that were transduced with SIT1 or GFP expression plasmid by a flow cytometry-based assay (Fig. 1f; Supplementary Fig. S9a). Strikingly, the maximum binding of WT or BA.5 RBD was dramatically reduced upon SIT1 overexpression (by 2.1 and 2.4 fold, respectively); their EC_50_ values were changed moderately, from 43.64 nM to 63.23 nM for WT RBD (1.4 fold), and from 16.99 nM to 18.41 nM for BA.5 RBD (1.1 fold) (Fig. 1g, h).

Next, we measured cell-surface ACE2 expression using an anti-ACE2 antibody by flow cytometry. We observed dramatically reduced cell-surface ACE2 levels in cells overexpressing SIT1 (Fig. 1i; Supplementary Fig. S9b). These data suggest that SIT1 overexpression reduces RBD binding to human cells mainly by reducing the amount of ACE2 proteins on the cell surface.

We also examined the subcellular localization of ACE2 and SIT1 in HEK293T cells using immunocytochemistry imaging. We observed strong co-localization of ACE2 and SIT1 (Fig. 1j), validating their interaction. Notably, overexpression of SIT1 led a substantial amount of ACE2 to be localized in the cytosol, in contrast to the predominant cell-surface localization of ACE2 in cells without SIT1 overexpression (Fig. 1j). These data suggest that SIT1 overexpression may reduce the level of cell surface ACE2 by trapping ACE2 in the cytosol.

The BA.4/BA.5 subvariants exhibit higher transmission advantage than Omicron BA.2, but the interaction between RBD and ACE2 seems quite consistent based on our structural analysis and binding assays. The putative reasons might be that the BA.4/BA.5 get an enhanced immune evasion capacity via the L452 substitutions and the F486V mutation^15^. We also demonstrate that overexpression of SIT1 decreases RBD binding to human cells by reducing cell-surface ACE2 levels possibly through restraining ACE2 in the cytosol. However, in contrast to SIT1, the overexpression of B^0^AT1 did not affect ACE2 subcellular localization nor the cell surface ACE2 levels and RBD binding (Supplementary Fig. S10). Since the structures of ACE2–B^0^AT1 and ACE2–SIT1 are quite similar (Supplementary Fig. S8), why and how SIT1 could trap ACE2 in the cytosol remains an interesting question. Additional proteins interacting with SIT1 may be involved in regulating ACE2 localization, which needs further investigation.

## Supplementary information

Supplementary information

## Data Availability

The structures of BA.2 RBD–ACE2–SIT1(PDB: 7Y75, whole map: EMD-33652, map focused on RBD–ACE2: EMD-33654, map focused on ACE2–SIT1: EMD-33655) and BA.5 RBD–ACE2–SIT1(PDB: 7Y76, whole map: EMD-33653, map focused on RBD–ACE2: EMD-33656, map focused on ACE2–SIT1: EMD-33657) have been deposited to the Protein Data Bank (http://www.rcsb.org) and the Electron Microscopy Data Bank (https://www.ebi.ac.uk/pdbe/emdb/), respectively. The other PDB and EMDB IDs can be found in Supplementary Table S1.
