# Supplementary material for: Structures of ACE2–SIT1 recognized by Omicron variants of SARS-CoV-2
Source: Cell Discov. 2022 Nov 16;8:123. doi: 10.1038/s41421-022-00488-x (PMC9667838; doi:10.1038/s41421-022-00488-x)
Supplement: Supplementary file 1 — Supplementary information [file 41421_2022_488_MOESM1_ESM.pdf]

Methods

Protein expression and purification

The cDNAs for full-length human SIT1 (accession number: NM_020208.3) and ACE2 (accession number: NM_001371415) were subcloned into pCAG respectively. An N-terminal FLAG tag was fused to SIT1, and 10×His tag was fused at the C-terminal of ACE2 using a standard two-step PCR. The receptor binding domains (RBDs) (319-541 a.a) of S protein from SARS-CoV-2 Omicron variant BA.2 and BA.5 were cloned into the pCAG vector (Invitrogen) with an N-terminal signal peptide of secreted luciferase and a C-terminal 6×His tag.

The recombinant protein was overexpressed using the HEK293F mammalian cells at 37℃ under 5% CO2 in a Multitron-Pro shaker (Infors, 130 rpm). When the cell density reached 2.0 $\times 10^6$ cells/mL, the plasmid was transiently transfected into the cells. All the plasmids used to transfect cells were prepared by GoldHi EndoFree Plasmid Maxi Kit (CWBIO).

To express the secreted RBD, about 1.5 mg of the RBD plasmid was premixed with 3 mg of polyethylenimines (PEIs) (Polysciences) in 50 mL of fresh medium for 15 mins before adding to cell culture. Cells were removed and medium was collected by centrifugation at 4000×g for 15 mins after sixty hours transfection.

The secreted RBD of S protein were purified by Ni-NTA affinity resin (Qiagen). The nickel resin loaded was rinsed with the wash buffer 1 containing 25 mM HEPES (pH 7.0), 500 mM NaCl and washed with wash buffer 2 containing 25 mM HEPES (pH 7.0), 150 mM NaCl and 30 mM imidazole. Protein was eluted by wash buffer 2 plus 270

mM imidazole. The Ni-NTA eluent of RBD was subjected to size-exclusion chromatography (Superose 6 Increase 10/300 GL, GE Healthcare) in PBS buffer (pH 7.4, gibco) with 0.04% Tween-20. The factions were stored at -80 °C.

To co-express SIT1 and ACE2, about 0.75 mg plasmid for SIT1 and 0.75 mg plasmid for ACE2 were premixed with 3 mg PEIs in 50 ml of fresh medium for 15 mins before adding to cell culture. The transfected cells were cultured for 48 hours before harvesting. For purification of the SIT1 and ACE2 complex, the cells were collected in buffer containing 25 mM HEPES, pH 7.0, 150 mM NaCl, and three protease inhibitors, aprotinin (1.3 μg/ml, AMRESCO), pepstatin (0.7 μg/ml, AMRESCO), and leupeptin (5 μg/ml, AMRESCO). The membrane fraction was solubilized at 4 ℃ for 2 hours with 1% (w/v) glyco diosgenin (GDN, Anatrace) and the cell debris was removed by centrifugation at 18,700 g for 45 mins. The supernatant was loaded to anti-FLAG M2 affinity resin (Sigma). After rinsing with the wash buffer 3 containing 25 mM HEPES, pH 7.0, 150 mM NaCl, and 0.01% GDN (w/v), the protein was eluted with wash buffer plus 0.2 mg/ml FLAG peptide. The eluent was further purified by Ni-NTA affinity resin (Qiagen). After elution with the wash buffer 3 supplemented with 300 mM imidazole, the eluent was then concentrated and incubated with BA.2/5 RBD at a molar ratio of about 1:2.4 for 30 mins. Then the protein mixture was subjected to size-exclusion chromatography (Superose 6 Increase 10/300 GL, GE Healthcare) in buffer containing 25 mM HEPES, pH 7.0, 150 mM NaCl and 0.01% GDN. The peak fractions were collected and concentrated for EM analysis.

Cryo-EM sample preparation and data acquisition

The protein of SIT1–ACE2–RBD complex was concentrated to 12 mg/mL and aliquots (3.3 μl) of the mixture were placed on glow-discharged holey carbon grids (Quantifoil Au R1.2/1.3), which were blotted for 3.0 s or 3.5 s and flash-frozen in liquid ethane cooled by liquid nitrogen with Vitrobot (Mark IV, Thermo Fisher Scientific). The cryo grids were transferred to a Titan Krios operating at 300 kV equipped with Gatan K3 Summit detector and GIF Quantum energy filter. Movie stacks were automatically collected using AutoEMation[1], with a slit width of 20 eV on the energy filter and a defocus range from -1.4 μm to -1.8 μm in super-resolution mode at a nominal magnification of 81,000 ×. Each stack was exposed for 2.56 s with an exposure time of 0.08 s per frame, resulting in a total of 32 frames per stack. The total dose rate was approximately 50 e^-/Å^2 for each stack. The stacks were motion corrected with MotionCor2[ref.2] and binned 2-fold, resulting in a pixel size of 1.087 Å/pixel. Meanwhile, dose weighting was performed[3]. The defocus values were further estimated with Gctf[4].

Data processing

Particles were automatically picked using Relion 3.0.6[ref.5-8] from manually selected micrographs. After 2D classification, good particles were selected and subjected to three cycles of heterogeneous refinement with C1 symmetry. The good particles were selected and subjected to homogeneous refinement with C2 symmetry. To further improve the map quality of ACE2-RBD interface, the particles were C2-symmetry expanded and re-extracted at the location of the interface between ACE2 and RBD. The re-extracted dataset was subject to several cycels of 3D classified and focused

refinement, resulting in a 3D reconstruction with better quality for ACE2–RBD interface. To further improve the map quality of the transmembrane domain of SIT1, the particles were C2-symmetry expanded and subjected to several cycels of 3D classified and focused refinement. The resolution was estimated with the gold-standard Fourier shell correlation 0.143 criterion[9] with high-resolution noise substitution[10].

Model building and structure refinement

The model building for the part of the SIT1–ACE2–RBD complex was accomplished with Phenix[11] and Coot[12]. The atomic model of the ACE2–B^0AT1–RBD (PDB ID: 6M17) was sequence-substituted to the SIT1, BA.2 RBD and BA.5 RBD in chainsaw and fitted into focused refined maps of transmembrane domain of SIT1 and ACE2–RBD interface using MDFF (molecular dynamics flexible fitting)[13]. Each residue was manually checked with Coot with the chemical properties taken into consideration during model building. Statistics associated with data collection, 3D reconstruction and model building are summarized in Supplementary Table S1.

The Bio-Layer Interferometry (BLI) assay

The bindings between ACE2 and Omicron subvariants RBD were performed using Octet Red96e (ForteBio), and the Wuhan-Hu-1 strain RBD was measured as well at the same time. The PD domain of ACE2 was biotinylated using biotinylation kit (Genemore, 1828M) and loaded to octet SA biosensor (Sartorius). The association and dissociation of ACE2 PD-coated biosensors with different concentrations of SARS-CoV-2 spike RBD were recorded using binding buffer (PBS pH 7.5, 0.04% Tween-20). Data were analyzed by Octet Data Analysis HT 12.0 software. Reference sample and

reference sensor were subtracted, and K_D values were analyzed using a 1:1 global fit model. Data were plotted using Prism V8.0 software (GraphPad).

Cell culture

HEK293T-ACE2 cells were obtained from Guoliang Zhang's lab as a gift. HEK293T-ACE2 and HEK293T cells were cultured in DMEM media (FI101-01, TransGen Biotech) supplemented with 10% fetal bovine serum (FS301-02,TransGen Biotech) and 1% penicillin-streptomycin (P301861,aladdin) at 37℃ and 5% CO2.

Transfection

Transient transfection of plasmids into HEK293T and HEK293T-ACE2 cells was performed using Hieff Trans™ Liposomal Transfection Reagent (40802ES02, YESEN) according to the manufacturer's instructions at a ratio of 1:2.5 (1 µg plasmid to 2.5 µl Hieff Trans™).

Measurement of RBD binding by flow cytometry

Cells were washed once with DPBS and dissociated with 0.05% EDTA. Then, cells were incubated with 3% BSA in DPBS for 20 minutes for blocking and transferred into 96-well plates. RBD proteins were diluted in 3% BSA to the appropriate concentrations and incubated with cells for 30 minutes on ice. Cells were then washed twice in 3% BSA in DPBS and incubated with APC-conjugated anti-His antibody (362605, Biolegend) at 1.5ul per 100ul cell suspension. Cells were washed twice and analyzed using a flow cytometer (BD FACSCanto SORP).

Immunocytochemistry

HEK293T cells were seeded on confocal plates coated with 0.5% Matrigel. Cells were

washed once with DPBS and fixed with 4% PFA for 20 mins, and then permeabilized in 0.5% Triton X-100 for 10 mins at room temperature. After washing with DPBS for three times, cells were blocked in blocking buffer (5% BSA in DPBS) for 1 hour at room temperature. Cells were then stained with primary antibodies diluted in blocking buffer overnight at 4℃. After that, cells were washed three times with DPBS, and then stained with fluorophore-conjugated secondary antibodies in blocking buffer for 1 hour. Cells were then washed with DPBS for three times and stained by DAPI. Images were acquired on a Nikon Confocal microscope (A1R+Symp64) using the confocal mode with 40X WI objective. Image analyses were preformed using Fiji (Version: 2.0.0-rc-69/1.52p).

Primary antibodies used in this study: rabbit anti-human ACE2 (1:200, ab272500, Abcam) and mouse anti-Flag (1:200, 109143-MM13, Sino Biological); Secondary antibodies used in this study: Alexa Fluor 488–conjugated Goat anti-rabbit IgG (1:400, D110090, Sangon Biotech) ; Alexa Fluor 555–conjugated Goat anti-mouse IgG (1:400, D110070, Sangon Biotech) and Alexa Fluor 647-conjugated Goat anti-rabbit IgG (1:400, D110078, Sangon Biotech).

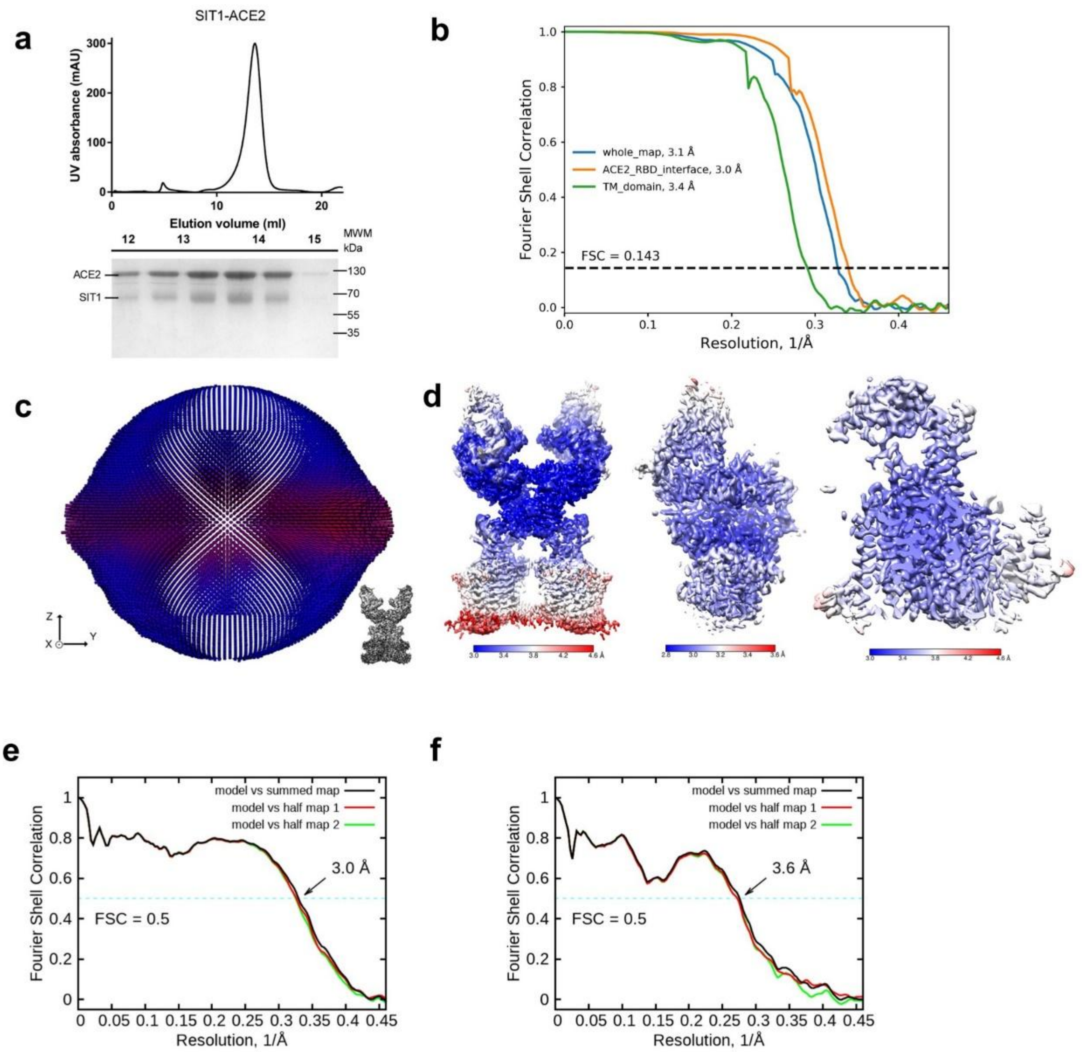

Fig. S1 Cryo-EM analysis of the SIT1–ACE2–BA.2 RBD ternary complex.

a Representative SEC purification of the SIT1–ACE2. SDS-PAGE was visualized by Coomassie blue staining. **b** Gold standard FSC curve of the Relion 3D reconstruction of the ternary complex. **c** Euler angle distribution of the ternary complex in the final 3D reconstruction. **d** Local resolution map for the 3D reconstruction of the ternary complex (left), the interface of ACE2–RBD (middle) and the transmembrane domain of SIT1 (right). **e** FSC curve of the refined model of ACE2–RBD subcomplex versus the overall structure that it is refined against (black); of the model refined against the first half map versus the same map (red); and of the model refined against the first half map versus the second half map (green). The small difference between the red and green curves indicates that the refinement of the atomic coordinates did not suffer from overfitting. **f** FSC curve of the refined model of the transmembrane domain of SIT1

versus the overall structure that it is refined against (black); of the model refined against the first half map versus the same map (red); and of the model refined against the first half map versus the second half map (green). The small difference between the red and green curves indicates that the refinement of the atomic coordinates did not suffer from overfitting.

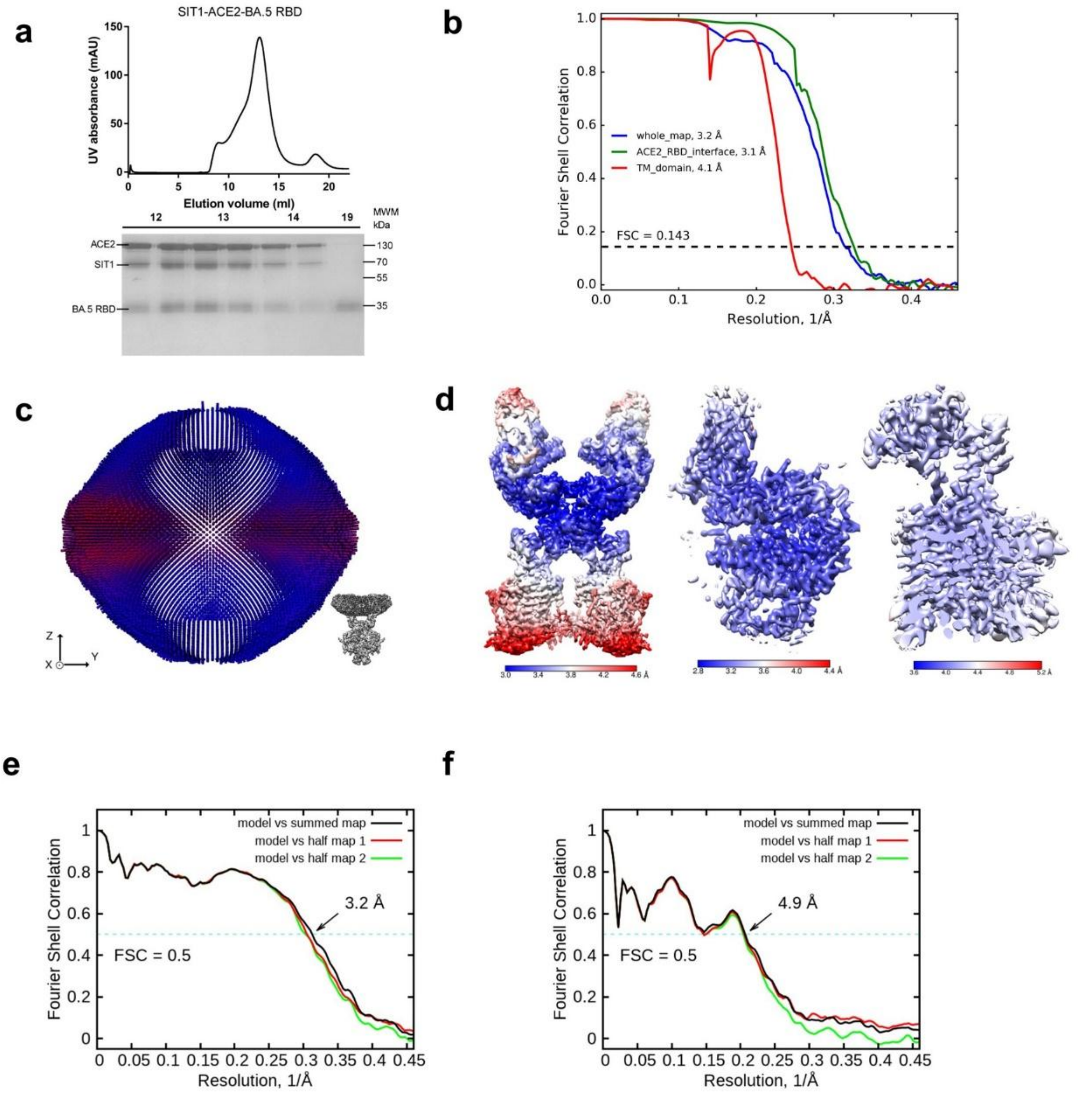

Fig. S2 Cryo-EM analysis of the SIT1–ACE2–BA.5 RBD ternary complex.

a Representative SEC purification of the SIT1–ACE2–BA.5 RBD. SDS-PAGE was visualized by Coomassie blue staining. **b** Gold standard FSC curve of the Relion 3D reconstruction of the ternary complex. **c** Euler angle distribution of the ternary complex in the final 3D reconstruction. **d** Local resolution map for the 3D reconstruction of the ternary complex (left), the interface of ACE2–RBD (middle) and the transmembrane domain of SIT1 (right). **e** FSC curve of the refined model of ACE2–RBD subcomplex versus the overall structure that it is refined against (black); of the model refined against the first half map versus the same map (red); and of the model refined against the first half map versus the second half map (green). The small difference between the red and green curves indicates that the refinement of the atomic coordinates did not suffer from overfitting. **f** FSC curve of the refined model of the transmembrane domain of SIT1 versus the overall structure that it is refined against

(black); of the model refined against the first half map versus the same map (red); and of the model refined against the first half map versus the second half map (green). The small difference between the red and green curves indicates that the refinement of the atomic coordinates did not suffer from overfitting.

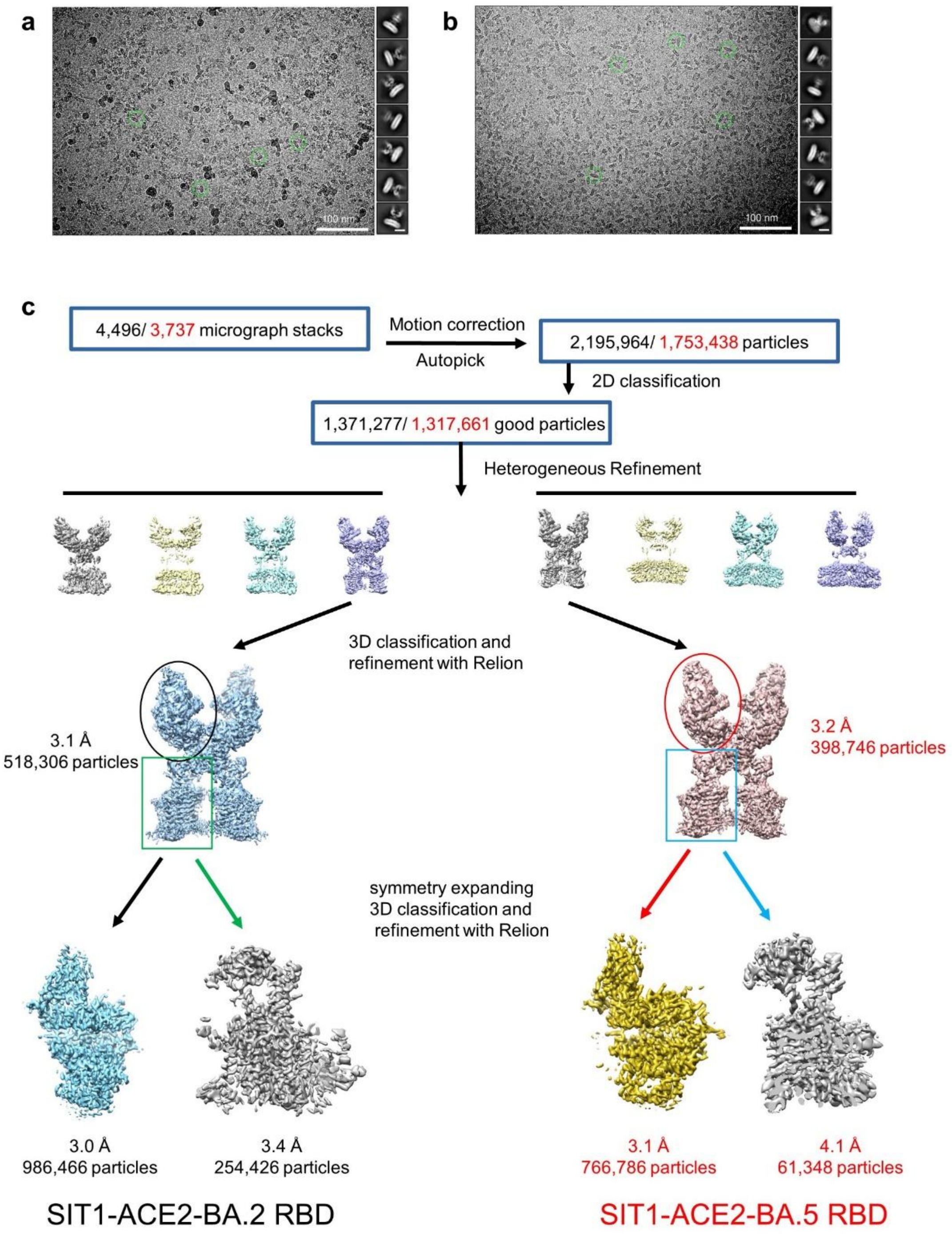

Fig. S3 Flowchart of cryo-EM data processing.

Please refer to the 'Data Processing' in Methods section for details.

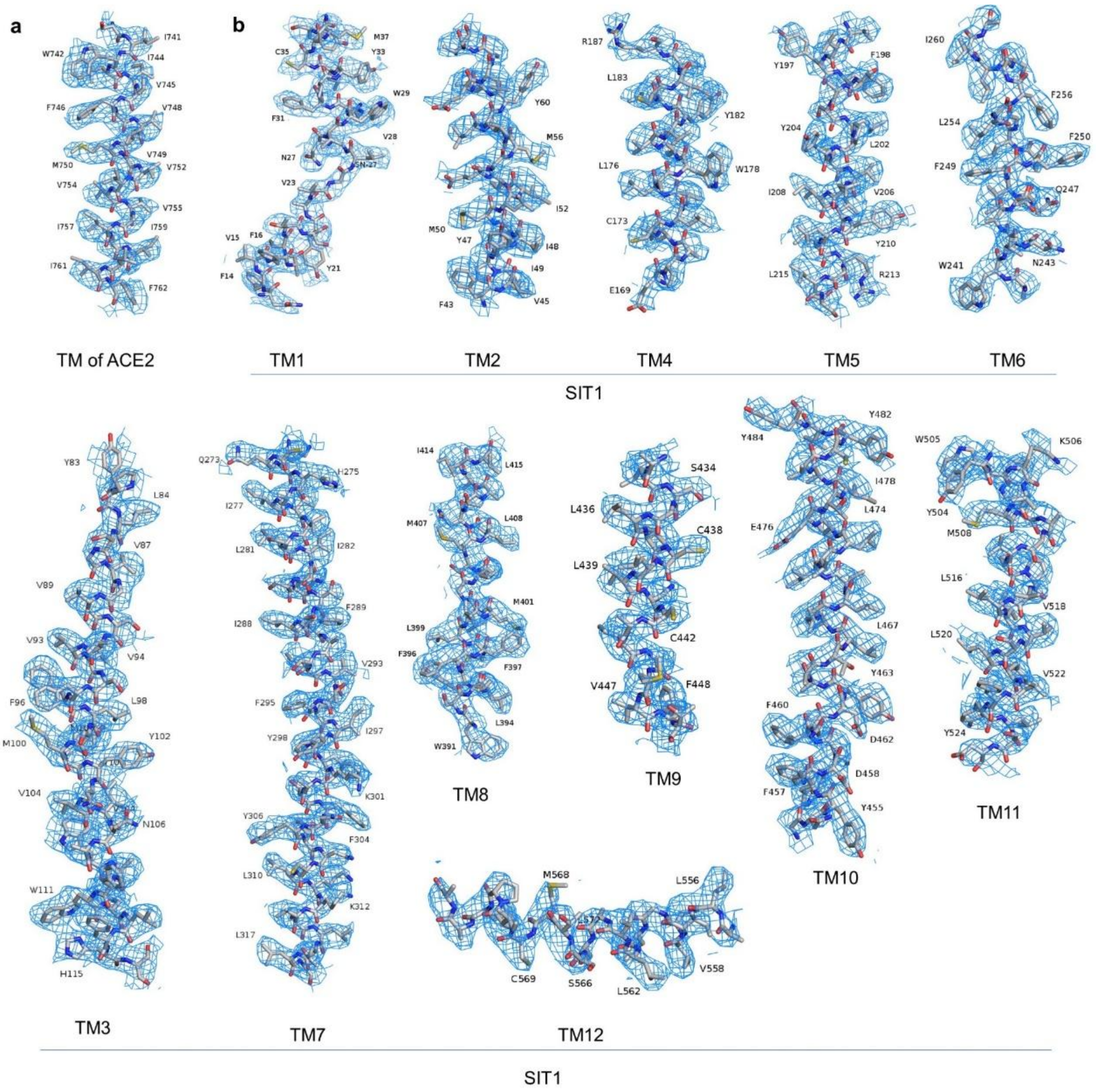

Fig. S4 Representative cryo-EM density maps of ACE2 and SIT1.

a Cryo-EM density map of transmembrane domain of ACE2 is shown at threshold of 7 σ. **b** Cryo-EM density map of transmembrane domain of SIT1 is shown at threshold of 7 σ.

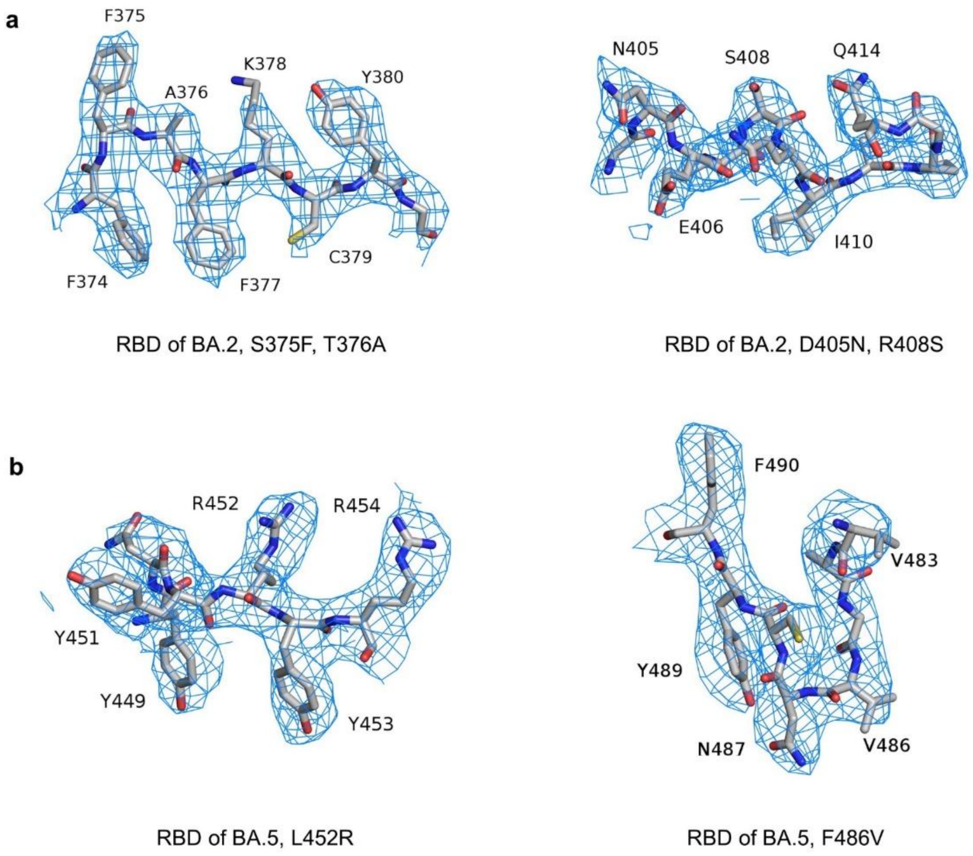

Fig. S5 Representative cryo-EM density maps of BA.2 RBD and BA.5 RBD.

a Cryo-EM density map of BA.2 RBD is shown at threshold of 7 σ. **b** Cryo-EM density map of BA.5 RBD is shown at threshold of 7 σ.

```
  WT RBD RVQPTESIVRFPNITNLCPFGEVFNATRFASVYAWNRKRISNCVA 363
BA.2 RBD RVQPTESIVRFPNITNLCPFDEVFNATRFASVYAWNRKRISNCVA 361
BA.5 RBD RVQPTESIVRFPNITNLCPFDEVFNATRFASVYAWNRKRISNCVA 359

  WT RBD DYSVLYNSASFSTFKCYGVSPTKLNDLCFTNVYADSFVIRGDEVR 408
BA.2 RBD DYSVLYNFAPFFAFKCYGVSPTKLNDLCFTNVYADSFVIRGNEVS 406
BA.5 RBD DYSVLYNFAPFFAFKCYGVSPTKLNDLCFTNVYADSFVIRGNEVS 404

  WT RBD QIAPGQTGKIADYNYKLPDDFTGCVIAWNSNNLDSKVGGNYNYLY 453
BA.2 RBD QIAPGQTGNIADYNYKLPDDFTGCVIAWNSNKLDSKVGGNYNYLY 451
BA.5 RBD QIAPGQTGNIADYNYKLPDDFTGCVIAWNSNKLDSKVGGNYNYRY 449

  WT RBD RLFRKSNLKPFERDISTEIYQAGSTPCNGVEGFNCYFPLQSYGFQ 498
BA.2 RBD RLFRKSNLKPFERDISTEIYQAGNKPCNGVAGFNCYFPLRSYGFR 496
BA.5 RBD RLFRKSNLKPFERDISTEIYQAGNKPCNGVAGVNCYFPLQSYGFR 494

  WT RBD PTNGVGYQPYRVVVLSFELLHAPATVCGPKKSTNLVKNKCVNF   541
BA.2 RBD PTYGVGHQPYRVVVLSFELLHAPATVCGPKKSTNLVKNKCVNF   539
BA.5 RBD PTYGVGHQPYRVVVLSFELLHAPATVCGPKKSTNLVKNKCVNF   537
```

Fig. S6 Sequence alignment of receptor binding domain in WT, BA.2 and BA.5 subvariants .

Sequence alignment of the RBD from indicated SARS–CoV–2 variants. The sequences are aligned using ClustalX. Identical residues are shaded gray.

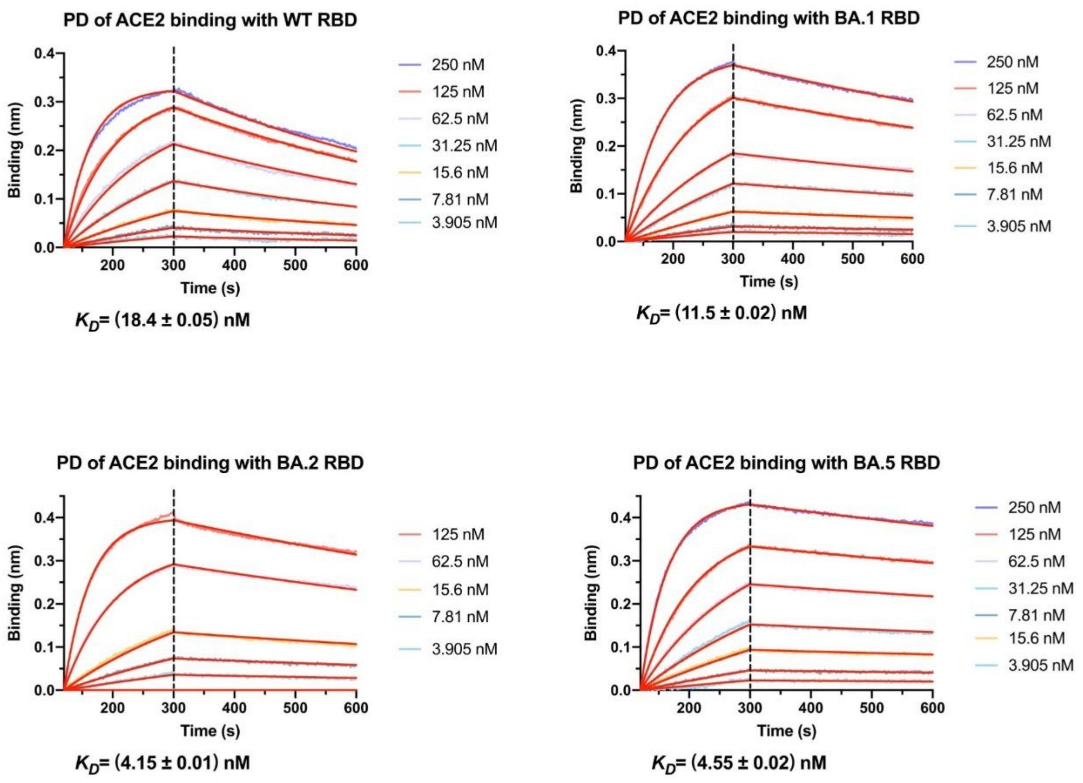

Fig. S7 Characterization of the binding affinity between ACE2 and Omicron subvariants BA.1, BA.2 and BA.4/BA.5 .

Binding of the peptidase domain (PD) of ACE2 to the receptor binding domain (RBD) of WT, BA.1, BA.2, BA.5 . The binding and K_D values were determined by BLI.

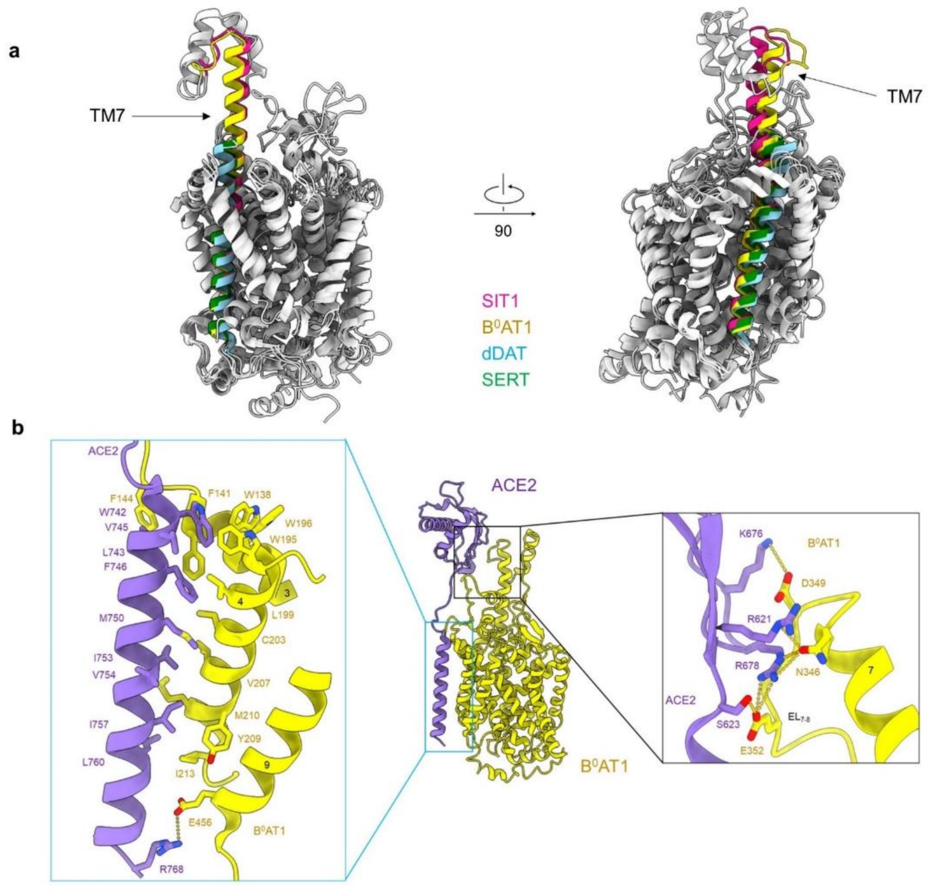

Fig. S8 The interaction between ACE2 and transporters in SLC6 family.

a The strunctural comparsion between SIT1, B^0AT1 (6M17), dDAT (4XPF) and SERT (5I71) shows the extended TM7 of SIT1 and B^0AT1 are necessary for the complex formation with ACE2. TM7 of SIT1, B^0AT1, dDAT and SERT are colored magenta, yellow, green and blue, respectively. **b** Structure of the transmembrane domain of ACE2 and B^0AT1 (6M17) is shown in the middle. ACE2 and B^0AT1 are colored purple and yellow, respectively. *Insets*: Enlarged views of the interface between the transmembrane helix of ACE2 and transmembrane helix 3,4,9 of B^0AT1 (left) and the interface between the extracellular loop of ACE2 and transmembrane helix 7 of B^0AT1 (right).

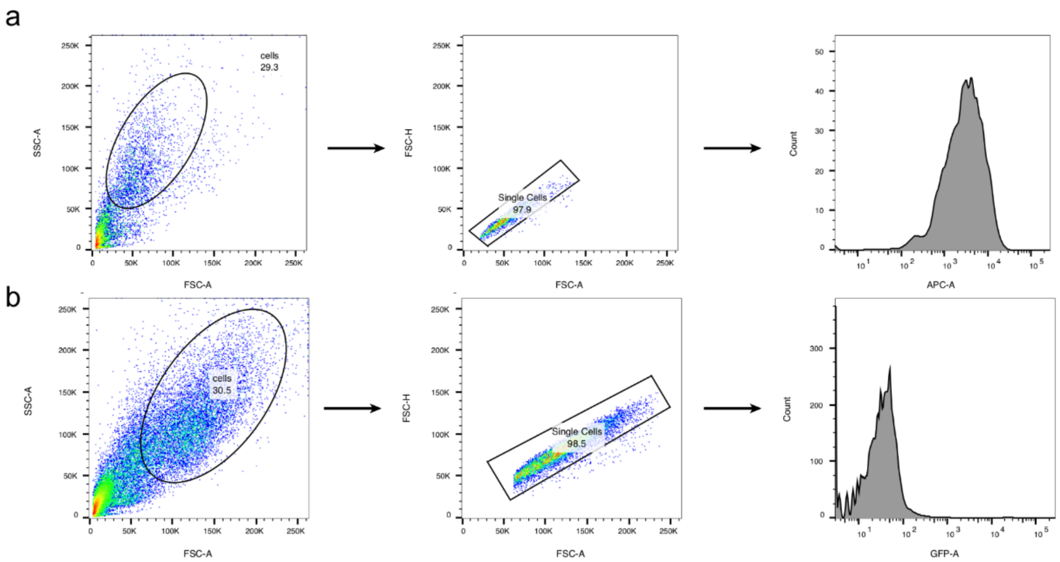

Fig. S9 Flow cytometry gating strategies, related to Fig. 1g,h (a) & Fig. 1i (b).

a Gating strategy for RBD-binding measurement. **b** Gating strategy for cell surface ACE2 measurement.

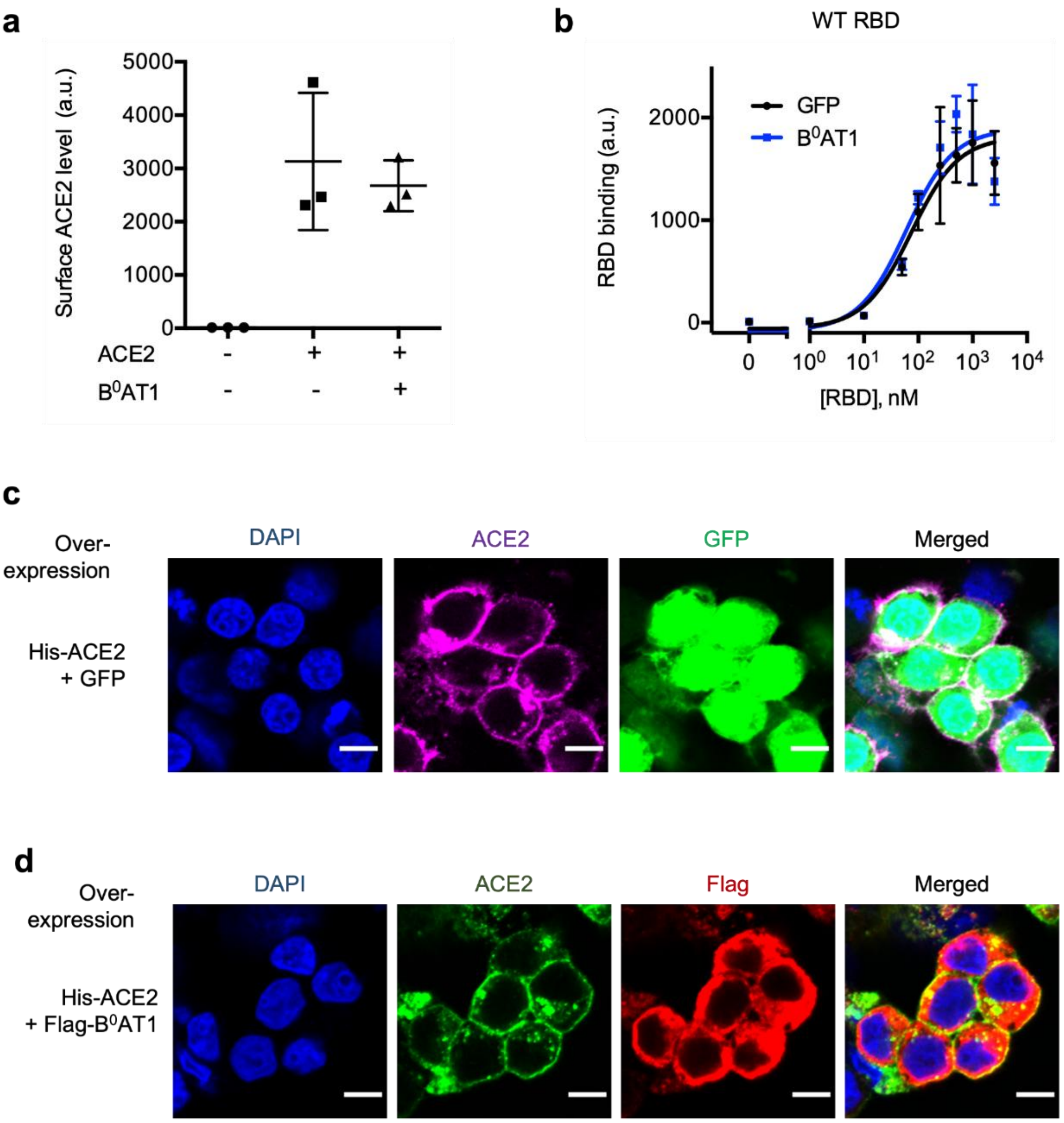

Fig. S10 RBD binding and ACE2 staining for cells overexpressing B^0AT1.

a Cell-surface ACE2 levels of cells overexpressing ACE2 and/or B^0AT1, measured by flow cytometry. Data are shown as means ± SD (*n* = 3). **b** WT RBD binding curves of HEK293-ACE2 cells overexpressing GFP (black) or B^0AT1 (blue). Data are shown as means ± SD (*n* = 3). **c** Representative immunostaining images of ACE2 for cells overexpressing His-tagged ACE2 and GFP. Scale bar, 10 μm. **d** Representative immunostaining images of ACE2 and B^0AT1 for cells overexpressing His-tagged ACE2 and Flag-tagged B^0AT1. Scale bar, 10 μm.

Table S1

**Data collection**		
EM equipment	Titan Krios (Thermo Fisher Scientific)	
Voltage (kV)	300	
Detector	Gatan K3 Summit	
Energy filter	Gatan GIF Quantum, 20 eV slit	
Pixel size (Å)	1.087	
Electron dose (e-/Å2)	50	
Defocus range (μm)	-1.4 ~ -1.8	
Sample	SIT1-ACE2-BA.2 RBD	SIT1-ACE2-BA.5 RBD
Number of collected micrographs	4,496	3,373
**3D Reconstruction**		
Software	Relion	
Sample	Overall	Overall
Number of used particles (Overall)	518,306	398,746
Resolution (Å)	3.1	3.2
Symmetry	C2	
Map sharpening B-factor (Å2)	-90	
**Refinement**		
Software	Phenix	
Cell dimensions		
a=b=c (Å)	313.056	
α=β=γ (˚)	90	
Model composition		
Protein residues	3,042	3,042
Side chains assigned	3,042	3,042
Sugar	38	38
Zn	2	2
Phospholipid	6	6
R.m.s deviations		
Bonds length (Å)	0.006	0.008
Bonds Angle (˚)	0.918	0.968

Ramachandran plot statistics (%)		
Preferred	93.80	93.1
Allowed	6.14	6.77
Outlier	0.07	0.13

Supplementary References:

1 Lei, J. & Frank, J. Automated acquisition of cryo-electron micrographs for single particle reconstruction on an FEI Tecnai electron microscope. *Journal of Structural Biology* **150**, 69-80, doi:https://doi.org/10.1016/j.jsb.2005.01.002 (2005).

2 Zheng, S. Q. *et al.* MotionCor2: anisotropic correction of beam-induced motion for improved cryo-electron microscopy. *Nature Methods* **14**, 331-332, doi:10.1038/nmeth.4193 (2017).

3 Grant, T. & Grigorieff, N. Measuring the optimal exposure for single particle cryo-EM using a 2.6 Å reconstruction of rotavirus VP6. *eLife* **4**, e06980, doi:10.7554/eLife.06980 (2015).

4 Zhang, K. Gctf: Real-time CTF determination and correction. *Journal of Structural Biology* **193**, 1-12, doi:https://doi.org/10.1016/j.jsb.2015.11.003 (2016).

5 Zivanov, J. *et al.* New tools for automated high-resolution cryo-EM structure determination in RELION-3. *eLife* **7**, e42166, doi:10.7554/eLife.42166 (2018).

6 Kimanius, D., Forsberg, B. O., Scheres, S. H. W. & Lindahl, E. Accelerated cryo-EM structure determination with parallelisation using GPUs in RELION-2. *eLife* **5**, e18722, doi:10.7554/eLife.18722 (2016).

7 Scheres, S. H. W. RELION: Implementation of a Bayesian approach to cryo-EM structure determination. *Journal of Structural Biology* **180**, 519-530, doi:https://doi.org/10.1016/j.jsb.2012.09.006 (2012).

8 Scheres, S. H. W. A Bayesian View on Cryo-EM Structure Determination. *Journal of Molecular Biology* **415**, 406-418, doi:https://doi.org/10.1016/j.jmb.2011.11.010 (2012).

9 Rosenthal, P. B. & Henderson, R. Optimal Determination of Particle Orientation, Absolute Hand, and Contrast Loss in Single-particle Electron Cryomicroscopy. *Journal of Molecular Biology* **333**, 721-745, doi:https://doi.org/10.1016/j.jmb.2003.07.013 (2003).

10 Chen, S. *et al.* High-resolution noise substitution to measure overfitting and validate resolution in 3D structure determination by single particle electron cryomicroscopy. *Ultramicroscopy* **135**, 24-35, doi:https://doi.org/10.1016/j.ultramic.2013.06.004 (2013).

11 Adams, P. D. *et al.* PHENIX: a comprehensive Python-based system for macromolecular structure solution. *Acta crystallographica. Section D, Biological crystallography* **66**, 213-221, doi:10.1107/s0907444909052925 (2010).

12 Emsley, P., Lohkamp, B., Scott, W. G. & Cowtan, K. Features and development of Coot.

Acta crystallographica. Section D, Biological crystallography **66**, 486-501, doi:10.1107/s0907444910007493 (2010).

13 Trabuco, L. G., Villa, E., Mitra, K., Frank, J. & Schulten, K. Flexible Fitting of Atomic Structures into Electron Microscopy Maps Using Molecular Dynamics. *Structure* **16**, 673-683, doi:https://doi.org/10.1016/j.str.2008.03.005 (2008).
